# RETRACTION: Expression of miR-155 in Serum Exosomes in Children with Epilepsy and Its Diagnostic Value

**DOI:** 10.1155/dim/9891815

**Published:** 2025-11-19

**Authors:** Disease Markers

RETRACTION: Y. Liu, G. Yu, Y. Ding, and Y. Zhang, “Expression of miR-155 in Serum Exosomes in Children with Epilepsy and Its Diagnostic Value,” *Disease Markers*, 2022, https://doi.org/10.1155/2022/7979500.

The above article, published online on 26 July 2022 in Wiley Online Library (wileyonlinelibrary.com), has been retracted by John Wiley & Sons Ltd. The retraction has been agreed due to significant errors raised on PubPeer in the Figures. Specifically:

• In Figure 2, the CD9 band 10^8^ Particles/mL exosome concentration appears to duplicated with the TNBS-treated TRP1A band in Figure 2a of [1] when rotated 180 degrees.
• In Figure 2, the GAPDH bands appear to be duplicates of the GAPDH bands in Figure 2a of [1].
• In Figure 2, the CD9 bands appear to be duplicates of the MTDH bands in [2].

The authors were given an opportunity to respond to the above concerns; however, no response was received.
